# High levels of high-density lipoprotein cholesterol may increase the risk of diabetic kidney disease in patients with type 2 diabetes

**DOI:** 10.1038/s41598-024-66548-2

**Published:** 2024-07-04

**Authors:** Huabin Wang, Junqi Wu, Meili Lin, Yifei Hu, Yongjun Ma

**Affiliations:** Department of Clinical Laboratory, Affiliated Jinhua Hospital, Zhejiang University School of Medicine, Renming Road, Jinhua City, 321000 Zhejiang Province China

**Keywords:** High-density lipoprotein cholesterol, Diabetic kidney disease, U-shaped association, Type 2 diabetes, Diabetes complications, Type 2 diabetes, Risk factors, Lifestyle modification, Nutritional supplements

## Abstract

Studies have indicated that low high-density lipoprotein cholesterol (HDL-C) level is an important risk factor for diabetic kidney disease (DKD) in patients with type 2 diabetes (T2D). However, whether higher HDL-C levels decrease the risk of developing DKD remains unclear. This study aimed to clarify the relationship between HDL-C levels and DKD risk in individuals with T2D in China. In total, 936 patients with T2D were divided into DKD and non-DKD groups. The association between HDL-C levels and DKD risk was evaluated using logistic regression analysis and restricted cubic spline curves adjusted for potential confounders. Threshold effect analysis of HDL-C for DKD risk was also performed. Higher HDL-C levels did not consistently decrease the DKD risk. Furthermore, a nonlinear association with threshold interval effects between HDL-C levels and the incidence of DKD was observed. Patients with HDL-C ≤ 0.94 mmol/L or HDL-C > 1.54 mmol/L had significantly higher DKD risk after adjusting for confounding factors. Interestingly, the association between high HDL-C levels and increased DKD risk was more significant in women. A U-shaped association between HDL-C levels and DKD risk was observed; therefore, low and high HDL-C levels may increase the DKD risk in patients with T2D.

## Introduction

In recent years, the incidence of diabetes mellitus has considerably increased, reaching approximately 536.6 million cases worldwide, and is projected to increase to 783.2 million by 2045^1^. Diabetic kidney disease (DKD) is one of the most common complications of diabetes, affecting approximately 20–40% of individuals^2,3^. In China, DKD has been identified as the leading cause of chronic kidney disease in adults, accounting for 26.7% of all renal disease cases^4^. Furthermore, DKD is widely recognized as a major contributor to end-stage renal disease and imposes a substantial health burden worldwide^5,6^. Despite efforts to achieve the recommended blood glucose and blood pressure targets, the residual risk of DKD remains high^7^, indicating an ongoing challenge in managing and preventing DKD progression. Therefore, given the profound impact of DKD, many researchers have increasingly focused on identifying clinical risk factors and biomarkers for the early detection, prevention, and intervention of DKD in recent decades^8–10^.

High-density lipoprotein cholesterol (HDL-C) is primarily synthesized in the liver, facilitating the cholesterol transportation from the extracellular tissues to the liver for metabolic processes. HDL-C has been considered a protective factor against cardiovascular events^11–13^. Similarly, some studies have indicated a strong association between low HDL-C levels and DKD development^7,14^. However, other studies have shown no significant difference in HDL-C levels between patients with and without DKD, indicating that HDL-C is not an independent risk factor for DKD^8,15,16^. Based on these conflicting results, we speculated that the relationship between HDL-C and DKD was not simply linear but rather nonlinear. Therefore, this study aimed to explore the relationship between HDL-C levels and the DKD risk in patients with type 2 diabetes (T2D).

## Materials and methods

### Study population

In this cross-sectional observational study, we randomly recruited patients with T2D who visited the Endocrinology Department at Jinhua Hospital, Zhejiang University School of Medicine, between September 2020 and July 2021. We included those aged ≥ 18 years with a diagnosis of T2D. The exclusion criteria were as follows: (1) patients diagnosed with kidney diseases other than DKD; (2) patients with severe acute infections and inflammation; (3) pregnant women; (4) patients with systemic immune diseases, such as systemic lupus erythematosus; (5) patients with tumors, severe cardiovascular diseases, or liver diseases; (6) patients with a rapid decline in kidney function in the short term; and (7) patients with missing pertinent clinical parameters. Based on the Cochran formula N = Z^2^ × p × (1 − p)/e^2^, the minimum required sample size was calculated to be 340, with a Z-value of 1.96 (corresponding to a 95% confidence level), a p-value representing the event occurrence rate of approximately 33%, and an e-value as the acceptable error margin of 5%. However, in this study, we actually utilized a sample size of 936. This number was based on the clinically available cases and reflected the distribution of the patient population, significantly exceeding the theoretical minimum sample requirement. This study followed the tenets of the Declaration of Helsinki and was approved by the Ethics Committee of Affiliated Jinhua Hospital, Zhejiang University School of Medicine (approval code: (Res) 2020-Ethical Review-200). Written informed consent was obtained from all participants.

### Data collection

All clinical information of the participants, including age, sex, height, weight, diastolic blood pressure (DBP), systolic blood pressure (SBP), diagnosis of hypertension, use of angiotensin-converting enzyme inhibitor (ACEI) and angiotensin II receptor blocker (ARB) medication, diabetes duration, and insulin use, was collected from the electronic medical record system. The laboratory indicators of the patients were collected from the Department of Clinical Laboratory. Fasting blood glucose, low-density lipoprotein cholesterol (LDL-C), HDL-C, triglycerides, total protein, serum albumin, uric acid, serum creatinine, blood urea nitrogen, urine albumin, and urine creatinine were measured using the Beckman Coulter AU5800 automatic biochemical analyzer (Beckman Coulter, Brea, CA, USA) and its original reagents. Glycated hemoglobin (HbA1c) was measured using a Bio-Rad D-100 analyzer (Bio-Rad, Hercules, CA, USA).

The body mass index (BMI) of the participants was calculated based on their height and weight. The urine albumin-to-creatinine ratio (ACR) was determined using the urine albumin and creatinine concentrations. The estimated glomerular filtration rate (eGFR) was determined using serum creatinine levels based on the Xiangya equation^17^. Renal dysfunction was identified when patients displayed an ACR > 30 mg/g and/or eGFR < 60 mL/min/1.73 m^2^.

### Statistical analysis

All statistical analyses were performed using R software (3.6.4 version) and SPSS 26.0. For continuous variables with a normal distribution, the mean and standard deviation were used for representation, whereas for continuous variables with a skewed distribution, the median and quartiles (Q1–Q3) were employed. Categorical variables are presented as counts and percentages (%). The study population was divided into DKD and non-DKD groups. Between-group comparisons were performed using Pearson’s chi-squared tests, unpaired t-tests, and Mann–Whitney tests, as applicable. HDL-C levels were divided into four groups based on quartiles: Q1: 0.40–0.96 mmol/L, Q2: 0.97–1.10 mmol/L, Q3: 1.11–1.31 mmol/L, and Q4: 1.32–6.27 mmol/L. Logistic regression analysis was performed to examine the relationship between HDL-C levels, as a continuous and a categorical variable, and the DKD risk in patients with T2D. A restricted cubic spline (RCS) curve was used to assess the nonlinear relationship between HDL-C levels and DKD risk. The R package “CatPredi” was utilized to identify the optimal cutoff points for the nonlinear association between HDL-C levels and DKD risk. Subsequently, based on the calculated optimal cutoff points, HDL-C was recategorized into three levels, and a stratified analysis was conducted to assess the relationship between different HDL-C levels and DKD risk in different subgroups. A two-tailed *p*-value < 0.05 was considered statistically significant.

## Results

The average age of the study population was 59.98 ± 12.85 years, with 48.08% (450/936) over the age of 60. Male participants accounted for 58.97% (552/936) of the population. A total of 55.02% (515/936) of the participants had been diagnosed with hypertension, and the average diabetes duration was 8.99 ± 7.36 years. The proportion of participants using ACEIs or ARBs was 24.15% (226/936). Notably, almost all individuals using ACEIs/ARBs had hypertension.

Among the 936 participants with T2D, the proportion of patients with DKD was 309 (33.01%). The major clinical characteristics of the study population are summarized in Table 1. Significant differences were observed in age, duration of diabetes, hypertension, BMI, ACEI/ARB use, SBP, triglycerides, uric acid, serum creatinine, and blood urea nitrogen levels between the DKD and non-DKD groups. However, no statistical differences were detected between the two groups in terms of sex, DBP, HbA1c levels, fasting blood glucose levels, or LDL-C levels. Notably, there was no significant statistical difference in HDL-C levels between the two groups (1.17 vs. 1.15 mmol/L, *P* = 0.384).

**Table 1:** Clinical characteristics of the study participants.

Variables	Non-DKD group (n = 627)	DKD group (n = 309)	*P*-value
Age (year)	58.31 ± 12.04	63.33 ± 13.73	< 0.001
Gender, n (%)			
Female	254 (40.51)	130 (42.07)	0.648
Male	373 (59.49)	179 (57.93)	
Diabetic duration (year)	7.71 ± 6.75	11.67 ± 7.84	< 0.001
Hypertension, n (%)			
No	347 (55.34)	74 (23.95)	< 0.001
Yes	280 (44.66)	235 (76.05)	
BMI	24.69 ± 3.73	25.26 ± 3.90	0.033
ACEI/ARB use, n (%)			
No	517 (82.45)	193 (62.46)	< 0.001
Yes	110 (17.54)	116 (37.54)	
Lipid lowering agents			
User of Fibrate, n (%)	19 (3.03)	17 (5.50)	0.064
User of Statin, n (%)	131 (20.89)	73 (23.62)	0.341
SBP (mmHg)	135 ± 17	144 ± 21	< 0.001
DBP (mmHg)	79 ± 11	79 ± 14	0.851
HbA1c (%)	8.16 ± 2.13	8.32 ± 2.17	0.276
Fasting blood glucose (mmol/L)	7.69 ± 2.72	7.76 ± 2.99	0.713
LDL-C (mmol/l)	2.94 ± 0.85	2.82 ± 1.01	0.07
HDL-C (mmol/l)	1.17 ± 0.34	1.15 ± 0.36	0.384
Triglyceride (mmol/l)	1.37 (1.01–1.95)	1.53 (1.08–2.14)	0.007
Total protein (g/L)	65.21 ± 5.18	64.86 ± 6.81	0.421
Serum albumin (g/L)	40.36 ± 3.54	38.26 ± 5.53	< 0.001
Uric acid (μmol/L)	291 (248–352)	348 (279–423)	< 0.001
Serum creatinine (μmol/L)	70.07 ± 13.40	101.92 ± 71.21	< 0.001
Blood urea nitrogen (mmol/l)	5.36 (4.46–6.39)	6.63 (5.43–8.54)	< 0.001
eGFR (ml/min/1.73 m^2^)	80.35 ± 9.31	69.10 ± 15.11	< 0.001
ACR (mg/g)	7.66 (4.46–12.46)	110.14 (47.16–495.94)	< 0.001

*DKD* Diabetic kidney disease, *BMI* Body mass index, *ACEI* Angiotensin converting enzyme inhibitor, *ARB* Angiotensin receptor blocker, *SBP* Systolic blood pressure, *DBP* Diastolic blood pressure, *HbA1c* Glycated hemoglobin, *LDL-C* Low-density lipoprotein cholesterol, *HDL-C* High-density lipoprotein cholesterol, *eGFR* estimated glomerular filtration rate, *ACR* Albumin-to-creatinine ratio, *T2D* Type 2 diabetes.

Overall, there was no statistically significant association between HDL-C level as a continuous variable and DKD risk in patients with T2D. However, after the categorization of HDL-C levels into four groups, compared with the lowest quartile of HDL-C (Q1), the DKD risk ratios (odds ratios [OR]) for Q2, Q3, and Q4 were 0.63 (0.43, 0.92), 0.50 (0.34, 0.73), and 0.62 (0.43, 0.91), with a P-value for trend of 0.003 (Table 2). Importantly, this decreasing trend was not consistently associated with increasing HDL-C quartiles (Q2, Q3, Q4) sequentially. Numerically, patients with the highest HDL-C levels (Q4) had slightly higher DKD risk compared with those with HDL levels in Q3. After adjusting for confounding factors (age, sex, BMI, SBP, DBP, HbA1c level, hypertension, and diabetes duration), this trend persisted, although the association was not statistically significant.

**Table 2:** The relationship of HDL-C with the risk of DKD in patients with T2D.

Variables	Univariate analyses		Multivariate analyses^#^	
	OR (95% CI)	*P* value	OR (95% CI)	*P* value
HDL-C (mmol/L)				
Continuous	0.83 (0.55, 1.26)	0.385	0.97 (0.60, 1.46)	0.902
Q1 (0.40–0.96)	1.0 (refference )	–	1.0 (refference )	–
Q2 (0.97–1.10)	0.63 (0.43, 0.92)	0.016	0.67 (0.44, 1.02)	0.060
Q3 (1.11–1.31)	0.50 (0.34, 0.73)	< 0.001	0.56 (0.36, 0.85)	0.007
Q4 (1.32–6.27)	0.62 (0.43, 0.91)	0.014	0.65 (0.42, 1.01)	0.054
*P* for trend	0.003		0.047	

^#^Adjusted for age, gender, body mass index, systolic blood pressure, diastolic blood pressure, glycated hemoglobin, hypertension and diabetic duration. *DKD* Diabetic kidney disease, *HDL-C* High-density lipoprotein cholesterol, *OR* Risk ratio, *T2D* Type 2 diabetes.

**Figure 1:**
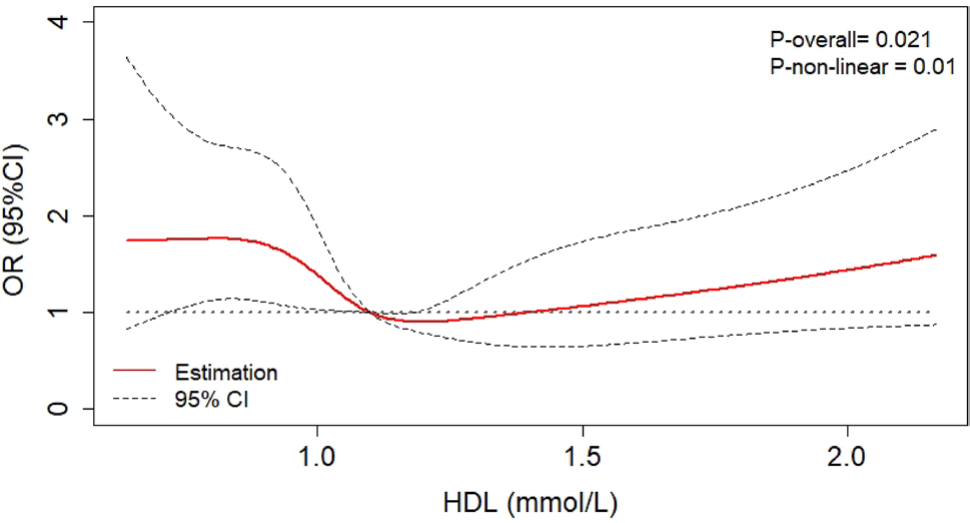
The RCS analysis based on logistic regression showed a U-shaped relationship between HDL-C levels and the risk of DKD in patients with T2D after adjustment for age, gender, body mass index, systolic blood pressure, diastolic blood pressure, glycated hemoglobin, hypertension and diabetic duration. DKD: diabetic kidney disease; RCS: restricted cubic spline; HDL-C: high-density lipoprotein cholesterol; OR: odds ratio; T2D: type 2 diabetes.

A U-shaped association between HDL-C levels and DKD risk in patients with T2D (P_nonlinear_ = 0.010) was observed after adjusting for potential confounders (Fig. 1). Based on the RCS curve, the HDL-C level was divided into three categories to calculate the optimal cutoff points. Finally, two threshold values of 0.95 and 1.54 mmol/L were selected. The threshold effects of HDL-C levels on DKD risk in patients with T2D were also assessed using logistic regression analysis (Table 3). After adjustment for the potential confounders, DKD risk in participants with HDL-C < 0.95 mmol/L and participants with HDL-C > 1.54 mmol/L relatively increased by about 77% and 128%, respectively, compared with that in the patients with 0.95 ≤ HDL-C ≤ 1.54 mmol/L. Importantly, patients with HDL-C > 1.54 mmol/L had the highest DKD risk (OR = 2.28 [1.35, 3.86], P = 0.002).

**Table 3:** Threshold effect analyses of HDL-C on the risk of DKD in patients with T2D.

Variables	Univariate analyses		Multivariate analyses^#^	
	OR (95% CI)	*P* value	OR (95% CI)	*P* value
HDL-C (nmol/L)				
0.95–1.54 mmol/L	1.0 (refference)	–	1.0 (refference)	–
< 0.95 mmol/L	2.08 (1.51, 2.87)	< 0.001	1.77 (1.23, 2.54)	0.002
> 1.54 mmol/L	2.02 (1.28, 3.20)	0.003	2.28 (1.35, 3.86)	0.002
*P* for trend	< 0.001		< 0.001	

^#^Adjusted for age, gender, body mass index, systolic blood pressure, diastolic blood pressure, glycated hemoglobin, hypertension and diabetic duration. *DKD* Diabetic kidney disease, *HDL-C* High-density lipoprotein cholesterol, *OR* Risk ratio, *T2D* Type 2 diabetes.

The stratified analysis revealed the varying effects of different HDL-C levels on DKD risk within different subgroups (Table 4). After adjusting for confounding factors, HDL-C > 1.54 mmol/L appeared to exert a stronger impact on DKD risk compared with HDL-C < 0.95 mmol/L in the three subgroups: age < 60 years (4.15 vs. 1.26), with hypertension (2.31 vs. 1.69), and without hypertension (2.37 vs. 2.03). Conversely, in the age > 60 years and female subgroups, HDL-C < 0.95 mmol/L exhibited a relatively stronger effect on DKD risk compared with HDL-C > 1.54 mmol/L. In this subgroup analysis, HDL-C > 1.54 mmol/L and HDL-C < 0.95 mmol/L showed the strongest impact on DKD risk in the female subgroup. However, in the male subgroup, no statistically significant association was observed between the categorization of HDL-C and DKD risk after adjusting for confounding factors (P > 0.05).

**Table 4:** The impact of HDL-C level on the risk of DKD in subgroups of patients with T2D.

Subgroups	Univariate analyses		Multivariate analyses^#^	
	OR (95% CI)	*P* value	OR (95% CI)	*P* value
Subjects with age ≤ 60 years				
0.95 ≤ HDL-C ≤ 1.54 mmol/L	1.0 (refference)	–	1.0 (refference)	–
HDL-C < 0.95 mmol/L	1.68 (1.04, 2.71)	0.034	1.26 (0.73, 2.19)	0.403
HDL-C > 1.54 mmol/L	2.99 (1.57, 5.70)	0.001	4.15 (1.84, 9.35)	0.001
*P* for trend	0.001		0.003	
Subjects with age > 60 years				
0.95 ≤ HDL-C ≤ 1.54 mmol/L	1.0 (refference)	–	1.0 (refference)	–
HDL-C < 0.95 mmol/L	2.77 (1.74, 4.41)	< 0.001	2.15 (1.29, 3.59)	0.003
HDL-C > 1.54 mmol/L	1.41 (0.73, 2.71)	0.305	1.47 (0.71, 3.08)	0.302
*P* for trend	< 0.001		0.012	
Female				
0.95 ≤ HDL-C ≤ 1.54 mmol/L	1.0 (refference)	–	1.0 (refference)	–
HDL-C < 0.95 mmol/L	3.64 (1.97, 6.70)	< 0.001	3.64 (1.81, 7.34)	< 0.001
HDL-C > 1.54 mmol/L	2.08 (1.16, 3.75)	0.014	2.65 (1.32, 5.33)	0.006
*P* for trend	< 0.001		< 0.001	
Male				
0.95 ≤ HDL-C ≤ 1.54 mmol/L	1.0 (refference)	–	1.0 (refference)	–
HDL-C < 0.95 mmol/L	1.71 (1.16, 2.53)	0.007	1.39 (0.90, 2.15)	0.141
HDL-C > 1.54 mmol/L	1.94 (0.92, 4.18)	0.082	2.30 (0.99, 5.35)	0.054
*P* for trend	0.012		0.078	
Subjects without hypertension				
0.95 ≤ HDL-C ≤ 1.54 mmol/L	1.0 (refference)	–	1.0 (refference)	–
HDL-C < 0.95 mmol/L	2.09 (1.14, 3.83)	0.017	2.03 (1.07, 3.82)	0.029
HDL-C > 1.54 mmol/L	2.02 (0.95, 4.31)	0.068	2.37 (1.05, 5.33)	0.038
*P* for trend	0.024		0.021	
Subjects with hypertension				
0.95 ≤ HDL-C ≤ 1.54 mmol/L	1.0 (refference)	–	1.0 (refference)	–
HDL-C < 0.95 mmol/L	1.88 (1.25, 2.81)	0.002	1.69 (1.08, 2.64)	0.022
HDL-C > 1.54 mmol/L	2.57 (1.33, 4.95)	0.005	2.31 (1.14, 4.69)	0.021
*P* for trend	0.001		0.010	

^#^Adjusted for age, gender, body mass index, systolic blood pressure, diastolic blood pressure, glycated hemoglobin, hypertension and diabetic duration; in each subgroup, the model was not adjusted for the stratification variable. *DKD* Diabetic kidney disease, *HDL-C* High-density lipoprotein cholesterol, *OR* Risk ratio, *T2D* Type 2 diabetes.

## Discussion

Our results demonstrated that in patients with T2D, the relationship between HDL-C levels and the incidence of DKD was U-shaped, with the low and high HDL-C level groups showing a significantly higher prevalence of DKD than the group with HDL-C levels between 0.95 and 1.54 mmol/L. Intriguingly, this relationship was more pronounced in women but was not statistically significant in men.

DKD is a chronic and detrimental complication of diabetes^7^. Despite the enhanced understanding of DKD in recent years and improved management of known significant risk factors, such as blood glucose and blood pressure, the residual risk of DKD remains high^18^, leading to more patients with DKD each year. In China, DKD has supplanted glomerulonephritis as the primary cause of newly diagnosed chronic kidney disease since 2011^19^. Therefore, it is imperative to identify modifiable risk factors. Patients with DKD not only face the risk of future kidney failure but also have a significantly increased risk of cardiovascular disease because of its significant association with cardiovascular risk^20–22^. In the past, HDL-C was known to be a protective factor against cardiovascular events. However, recently, more observational studies have shown that high levels of HDL-C are associated with an elevated risk of cardiovascular events and all-cause mortality^23–25^. Liu et al.^26^ conducted a study on individuals diagnosed with coronary heart disease. They found that individuals with low or high HDL-C levels had an increased risk of all-cause and cardiovascular mortality compared with individuals with normal HDL-C levels. Nevertheless, there is controversy in the current research regarding the relationship between HDL-C levels and DKD risk in patients with T2D. In addition, the effect of elevated HDL-C levels on the risk of developing DKD remains unclear.

To further explore the association between HDL-C levels and the incidence of DKD in patients with T2D, we conducted a study involving 936 participants to systematically analyze the explicit relationship between HDL-C levels and DKD risk. Our observations address an important knowledge gap in this field. In the present study, there was no significant difference in the HDL-C levels between the DKD and non-DKD groups as continuous variables. This result is consistent with those of other studies^15,16,27^. However, other studies showed significant differences in HDL-C levels between the two groups, with the DKD group having significantly lower HDL-C levels than the non-DKD group^9,28^. The reasons for the discrepancy in these research results might include differences in the study populations and variations in the methods used to measure HDL-C levels. When HDL-C was categorized into four quartiles, our findings indicated that, compared with the lowest quartile of HDL-C, the second, third, and fourth quartiles had a lower risk of DKD, with the lowest risk observed in the third quartile. As a result, the hypothesis of a nonlinear U-shaped relationship between HDL-C levels and DKD risk was proposed, which was subsequently confirmed by the RCS and stratified analyses in this study.

A previous investigation^7^ based on Italian diabetes centers analyzed the relationship between plasma HDL-C levels and the prevalence of DKD in patients with T2D. They found that low HDL-C level was an independent risk factor for the prevalence of DKD, as evidenced by associations with low eGFR, eGFR reduction, and albuminuria. This relationship remained statistically significant when analyzing HDL-C concentration as a continuous variable (each 10 mg/dL increase) and exhibited only attenuation following adjustment for numerous confounding factors in the multivariate analysis (P < 0.05)^7^. The discrepancy between the data and ours might be explained by the different study populations and the differential lipid metabolism profiles within the two study populations (1.35 ± 0.39 vs. 1.16 ± 0.35 mmol/L for the average HDL-C levels). Regardless of the case, the U-shaped relationship between HDL-C levels and the prevalence of DKD in patients with T2D provided a reasonable explanation for low levels of HDL-C being an important risk factor for DKD; however, extremely high levels of HDL-C showed diminished protective effects against DKD.

The U-shaped association between HDL-C levels and DKD risk indicates that low and high HDL-C levels can increase DKD risk. First, low HDL-C levels are associated with increased oxidative stress and inflammation, both of which play crucial roles in the pathogenesis of renal damage in patients with diabetes^29^. Specifically, low HDL-C levels may impair the reverse cholesterol transport mechanism, leading to lipid accumulation in renal cells and thereby exacerbating kidney injury^29^. Additionally, low HDL-C levels may reflect an overall dyslipidemic condition, including elevated triglyceride and LDL-C levels, which increase vascular damage risk and promote the progression of microvascular complications in diabetes^30^. Conversely, very high HDL-C levels may not provide cardiovascular protection and even pose risks^31^. This apparent contradiction may be because of functional impairments of HDL-C at high concentrations. This can include reduced efficacy of reverse cholesterol transport and anti-inflammatory activities, potentially increasing the risk of cardiovascular and renal complications^31,32^. Genetic and environmental factors may play significant roles in this complex relationship^33,34^. For instance, genetic variations affecting HDL-C metabolism and the impact of chronic conditions such as diabetes can alter the protective effects of HDL-C. Further research is required to elucidate the underlying mechanisms.

Intriguingly, the increased DKD risk associated with high HDL-C levels (> 1.54 mmol/L) was not confirmed in the male or over 60 years of age subgroups. A study on the relationship between high HDL-C levels and the risk of cardiovascular events yielded similar results, showing that high HDL-C levels have different effects on the risk of cardiovascular events in men and women^31^. These findings might be explained by sex-based differences in cholesterol. For instance, well-documented evidence shows that women typically exhibit higher HDL-C levels than men^31,35^, and estrogen can elevate HDL-C^36^. The reasons for the differential impact of high HDL-C (> 1.54 mmol/L) on DKD risk in the two age subgroups remain unclear; it may be associated with alterations in lipid metabolism among individuals over 60 years of age.

This study has several limitations. First, the number of participants at the higher end of the HDL-C concentration spectrum was relatively small. Second, not all the factors influencing HDL levels were considered comprehensively. Specifically, variables such as obesity, dietary habits, sedentary lifestyle, alcohol consumption, and genetic diseases were not thoroughly controlled for and analyzed, potentially introducing bias into our results. This study did not systematically evaluate the impact of drugs known to affect HDL-C levels. Beta-blockers, thiazide diuretics, androgens, and anabolic steroids, known to potentially decrease HDL-C levels, were not included in our analysis. Conversely, drugs, such as niacin and PCSK9 inhibitors, known to increase HDL-C levels, were not systematically assessed. This variability in drug effects could have significantly affected HDL-C levels, thereby affecting the DKD risk assessment. Additionally, the potential effects of fluctuating estrogen levels during menopause and thyroid dysfunction on HDL-C levels were not addressed. In particular, hypothyroidism may cause abnormal lipid metabolism and lower HDL-C levels. This might have also contributed to bias in our findings. Finally, as this was a cross-sectional observational study, the findings could not robustly demonstrate nonlinear associations between HDL-C levels and DKD risk in patients with T2D. This study also did not conclusively prove a causal relationship between elevated HDL-C levels and an increased DKD risk. This preliminary study provided evidence of a U-shaped association between HDL-C levels and DKD risk, highlighting the importance and necessity of planning and conducting a prospective confirmatory study with a sufficiently large, randomly selected sample.

## Conclusion

Although HDL-C is generally considered a cardiovascular protective factor, at very high levels, this protective effect does not seem to hold true and may be associated with an increased DKD risk. Our findings suggest that a low HDL-C level increases the DKD risk, and HDL-C levels greater than 1.54 mmol/L are significantly associated with an increased DKD risk, especially in women. These findings may provide guidance for managing blood lipids to prevent and treat DKD in patients with T2D.

### Ethics approval and consent to participate

The study was conducted according to the guidelines of the Declaration of Helsinki, and approved by the Ethics Committee of Affiliated Jinhua Hospital, Zhejiang University School of Medicine (approval code: (Res) 2020-Ethical Review-200). Written informed consent was obtained from all participants.

## Data Availability

The data underlying this article will be shared on reasonable request to the corresponding author.
